# Effect of Ionic Liquid Surfactants on Coal Oxidation and Structure

**DOI:** 10.1155/2019/1868265

**Published:** 2019-04-22

**Authors:** Weiqing Zhang, Shuguang Jiang, Tong Qin, Jianfeng Sun, Chaowei Dong, Qiang Hu

**Affiliations:** ^1^State Key Laboratory of Coal Resources and Safe Mining, China University of Mining & Technology, Xuzhou 221116, Jiangsu, China; ^2^School of Safety Engineering, China University of Mining & Technology, Xuzhou 221116, Jiangsu, China; ^3^Department of Mining Engineering, Luliang University, Luliang 033000, Shanxi, China; ^4^School of Mines, China University of Mining & Technology, Xuzhou 221116, Jiangsu, China

## Abstract

The effects of six ionic liquids with surfactant property (1-hydroxyethyl-3-methyl imidazolium bis(trifluoromethylsulfonyl)imide ([HOEtMIm][NTf_2_]), 1-hydroxyethyl-3-methyl imidazolium tetrafluoroborate ([HOEtMIm][BF_4_]), 1-dodecyl-3- methyl imidazolium bromide ([C_12_MIm]Br), 1-tetradecyl-3- methyl imidazolium bromide ([C_14_MIm]Br), trioctyl methyl ammonium chloride ([N_8,8,8,1_])Cl, and tetraethyl ammonium chloride ([N_2,2,2,2_]Cl)) on the oxidation characteristics and functional groups of coal were studied by means of critical micelle concentration, surface tension, thermogravimetric analysis, temperature-programmed oxidation, and Fourier transform infrared spectroscopy (FTIR) measurements. The lower critical micelle concentration for the ionic liquids except the [N_2,2,2,2_]Cl suggests the favorable surface activity of these ionic liquids. The surface activities of [N_8,8,8,1_]Cl, [C_14_MIm]Br, [C_12_MIm]Br, and [HOEtMIm][NTf_2_] were high, while that of [N_2,2,2,2_]Cl was relatively lower. The thermal stabilities of [HOEtMIm][NTf_2_] and [HOEtMIm][BF_4_] were high, while those of [N_8,8,8,1_]Cl and [N_2,2,2,2_]Cl were lower. The oxidation activities of ionic liquid-mixed coals were weakened to different degrees except [N_8,8,8,1_]Cl-mixed coal, because of the poor thermal stability and decomposition of [N_8,8,8,1_]Cl accelerating the coal oxidation. The other five ionic liquids were suitable for inhibiting coal oxidation, particularly the [HOEtMIm][BF_4_] and [HOEtMIm][NTf_2_] with higher inhibition rate, longer inhibition time, and also better thermal stabilities. The activation energy results further confirmed such inhibition effect. The functional group results showed that treatment of ionic liquids on coal can change the contents of hydrogen bonds, aliphatic groups, and aromatic groups in coal. It was inferred that the [HOEtMIm][BF_4_], [HOEtMIm][NTf_2_], and [C_14_MIm]Br were more effectively to affect coal structure and decrease coal oxidation activity.

## 1. Introduction

The property of low temperature oxidation of coal can result in coal spontaneous combustion, which seriously threatens the safety and sustainable development of coal industry. At present, the study of inhibition agents to change the oxidation activity of coal becomes the hot spot of coal spontaneous combustion control, including the inorganic salts [1, 2] and organic matters [3, 4]. These chemical inhibitors show favorable inhibition effects on low temperature oxidation of coal.

Ionic liquids (ILs), as the hot spots in the field of green chemistry, consist of bulky and asymmetric organic cations and organic or inorganic anions [5, 6]. ILs have melting point below 100°C, negligible volatility, nonflammability, and excellent dissolving and swelling capacity to coal [7, 8]. Such capacity can influence the coal structure and change the coal oxidation property. Wang and Zhang et al. found that imidazolium-based ILs can partially change the oxygen-containing and aliphatic functional groups in coal and affect the oxidation properties of the coal [9–12]. Zhang et al. concluded that the phosphonium-based ILs can affect coal oxidation activity and inhibit coal oxidation process [13]. These results showed that ILs can affect coal microstructure as well as change coal oxidation activity.

The interaction between coal and inhibition agents is solid-liquid surface contact so that the inhibition agents with surface activity can interact with coal surface better and promote the inhibition effect. The surface tension of ILs is normally 21–60 mN/m [14, 15], which is lower than that of water (72 mN/m) so that the ILs with surface activity may promote the interaction of coal and ILs and better inhibit coal oxidation. Our previous research investigated the effect of some imidazolium-based and phosphonium-based ILs on coal wettability and microcosmic structure [16], indicating the wetting action of these ILs and the effect on functional groups of coal.

Herein, the authors further analyzed the effect of the typical ILs with surfactant property on coal oxidation and structure to provide more evidence for searching new materials which can significantly weaken the coal oxidation activity.

## 2. Experimental

### 2.1. Coal Sample and IL Surfactants

The coal sample was bituminous coal according to the China Standard GB/T 5751–2009. The moisture, ash, and volatility contents of the sample on the air-dry basis were 3.34, 11.62, and 31.83%, respectively.

The IL surfactant samples included four imidazolium-based ILs of 1-hydroxyethyl-3-methylimidazolium bis(trifluoromethylsulfonyl)imide ([HOEtMIm][NTf_2_]), 1-hydroxyethyl-3-methylimidazolium tetrafluoroborate ([HOEtMIm][BF_4_]), 1-dodecyl-3-methylimidazolium bromide ([C_12_MIm]Br), and 1-tetradecyl-3-methylimidazolium bromide ([C_14_MIm]Br) and two quaternary ammonium ILs of tricaprylmethylammonium chloride ([N_8,8,8,1_]Cl) and tetraethylammonium chloride ([N_2,2,2,2_]Cl). These ILs were purchased from Lanzhou GreenChem ILS, LICP, CAS (China) and used as received.

### 2.2. Sample Preparation

The concentration of the six IL surfactant solutions was 20% in distilled water (w/v), which is the inhibition concentration usually used in coal spontaneous combustion suppression study.

The coal sample was ground in a mortar, sieved to a particle size of 74 *μ*m, and then vacuum-dried in an oven at 27°C for 48 h. The dried particulate coal (∼5 g) was vigorously mixed with the six IL surfactant solutions (∼3 ml) separately. The mixed coal was vacuum-dried in oven at 27°C until the weight of the mixture unchanged, which was named as IL-mixed coal (IL-mc).

Part of the IL-mixed coal samples were washed with distilled water and filtered to separate the coal until the filtrate was transparent and neutral. Then, the washed coal was dried in a vacuum oven at 27°C until the weight of the coal samples unchanged, which was named as IL-treated coal (IL-tc).

In addition to the IL-mixed coals and IL-treated coals, a sample of the untreated particulate coal was washed only with distilled water to enable a comparison to be made with the IL-mixed coals and IL-treated coals; this sample was denoted as IL-untreated coal (IL-untc).

### 2.3. Experimental Procedures

The critical micelle concentration (CMC) of the six ILs was measured by ultraviolet-visible (UV-Vis) spectrophotometry (Thermo Biomate 3S) using a solvatochromic dye fluorescein. One drop of a 100 mL anhydrous ethanol solution containing 0.1 g of fluorescein was added in the cuvette for the experiments. The UV-Vis spectra were recorded between 200 and 600 nm with a spectral bandwidth of 1 nm.

The surface tensions of the six IL solutions were tested by the ring tear-off method using a Force Tensiometer (K100, KRÜSS GmbH, Germany).

The thermal stabilities of the six ILs were tested by the thermogravimetric analysis (TGA) using a thermogravimetric analyzer (Diamond TG/DTA 6300, PerkinElmer, UK) in dry air flow of 50 cm^3^/min at a heating rate of 5°C/min over the temperature range 30–800°C.

The oxidation properties of the IL-untc and six IL-mcs were measured using a temperature-programmed (TP) testing system [17] with 3 g coal samples in dry air flow of 20 cm^3^/min at a heating rate of 2°C/min over the temperature range 30–200°C. The gaseous products were analyzed by an indicator gas analysis system including a high-precision CO sensor, an O_2_ sensor, and a signal processing module.

The functional groups of the IL-untc and six IL-tcs were subjected to Fourier-transform infrared spectroscopy (FTIR) measurement. FTIR spectra were recorded between 3800 and 400 cm^−1^ and were accumulated for 32 scans at a resolution of 4 cm^−1^ on a FTIR spectroscope (Vertex 80v, Bruker, Germany).

## 3. Results and Discussion

### 3.1. CMC Results of the ILs

**Figure 1:**
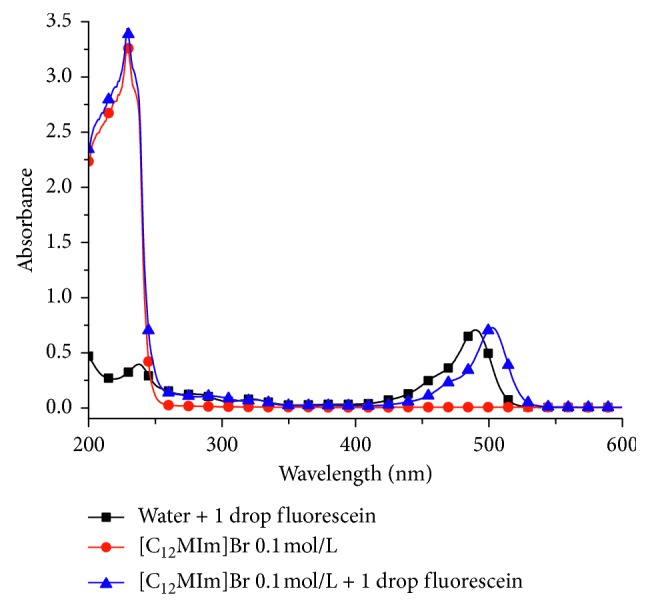
UV-Vis spectrum and the maximum absorption wavelength for fluorescein and/or [C_12_MIm]Br at 0.1 mol/L.

The CMC is a key characteristic for the IL surfactants. The surface tension changes strongly with the concentration changing before the CMC, then remains relatively constant after the CMC. The determination of the ILs CMC was based on the changes of the wavelength of the absorption maximum (*λ*_max_) [18–20], which is a semiquantitative method.  Figure 1 shows the *λ*_max_ of [C_12_MIm]Br at 0.1 mol/L (∼230 nm) and fluorescein (∼490 nm).

**Figure 2:**
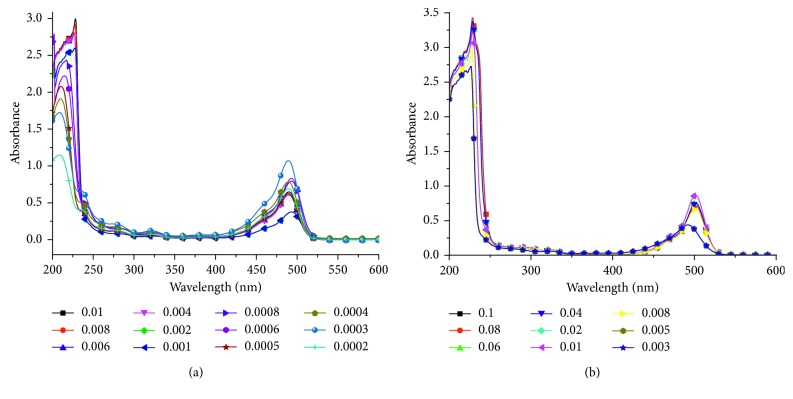
UV-Vis spectra for [HOEtMIm][NTf_2_] and [C_12_MIm]Br solutions at different concentrations (mol/L) containing fluorescein as fluorescence probe. (a) UV-Vis spectra for [HOEtMIm][NTf_2_] concentrations. (b) UV-Vis spectra for [C_12_MIm]Br solutions.

The *λ*_max_ changed suddenly before and after the CMC, such as the *λ*_max_ of [HOEtMIm][NTf_2_] changed from 217 nm at 0.0008 mol/L to 227 nm at 0.001 mol/L, and remained relatively unchanged with the concentration increasing (Figure 2(a)). The results provided the CMC value of [HOEtMIm][NTf_2_] was 0.001 mol/L. Similarly, the *λ*_max_ belonged to fluorescein changed from 490 nm at 0.005 mol/L [C_12_MIm]Br to 500 nm at 0.008 mol/L [C_12_MIm]Br and remained unchanged with the increasing concentration (Figure 2(b)). Such results gave the CMC value of [C_12_MIm]Br was 0.008 mol/L.  Table 1 lists the CMC values of the six ILs surfactants. The CMCs were found to be from 1 to 10 mM for the ILs except the [N_2,2,2,2_]Cl. This suggests the favorable surface activity of these ILs. Further, the concentration 20%, which was the inhibitor concentration usually used in coal spontaneous combustion suppression, was higher than the six CMCs so that the IL solutions (20%) used below can play both inhibitor and surfactant roles.

### 3.2. Surface Tension Results of the ILs

The surface tension results of the ILs solutions are shown in Table 2.

Generally, the critical surface tension of coal is 45 mN/m so that when the surface tension of ILs is lower than 45 mN/m, the ILs can wet coal surface [21].  Table 1 shows that the surface tension of the distilled water was 72.55 mN/m, while the surface tensions of the six IL solutions were lower than that of distilled water, indicating the high surface activity of the ILs. The lowest surface tension was that of [N_8,8,8,1_]Cl and then were the two typical IL surfactants [C_12_MIm]Br and [C_14_MIm]Br. The surface tension of [HOEtMIm][NTf_2_] was nearly the same as that of the two typical IL surfactants. The maximum surface tension was from IL [N_2,2,2,2_]Cl, indicating its low surface activity. The surface tension of [HOEtMIm][NTf_2_] was lower than that of [HOEtMIm][BF_4_] because of the more obvious amphiphilic features of [HOEtMIm][NTf_2_] caused by the stronger electronegativity of anion [NTf_2_]^−^ than that of [BF_4_]^−^. The surface tension of [C_14_MIm]Br was slightly lower than that of [C_12_MIm]Br because of the bigger volume of the cationic hydrophobic group [C_14_MIm]^+^ with longer alkyl side chain. In the same way, the surface tension of [N_8,8,8,1_]Cl was far lower than that of [N_2,2,2,2_]Cl because of the bigger volume of [N_8,8,8,1_]^+^.

In summary, the surface activity of the six IL solutions from strong to weak was [N_8,8,8,1_]Cl > [C_14_MIm]Br > [C_12_MIm]Br > [HOEtMIm][NTf_2_] > [HOEtMIm][BF_4_] > [N_2,2,2,2_]Cl.

### 3.3. Thermal Stability Results of the ILs

**Figure 3:**
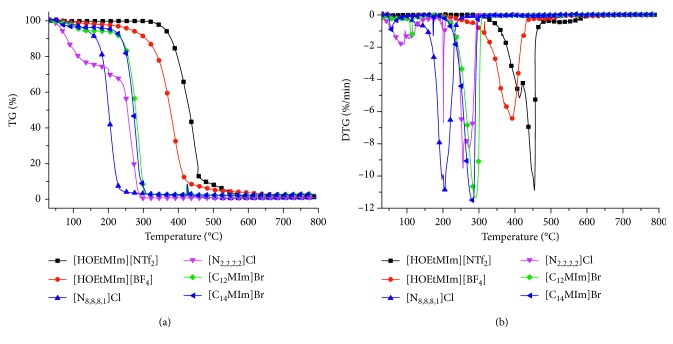
TG-DTG results of ILs. (a) TG. (b) DTG.

Figure 3 shows the TG and DTG results of the six ILs. According to the results, the ILs with better thermal stability can be chosen and further used for inhibition agent.

From Figure 3(a), the mass loss of [N_2,2,2,2_]Cl occurred firstly at 50°C and increased to ∼25% mass loss at 200°C. Then, the mass lost quickly and nearly completely lost at ∼280°C. It can be deduced that the [N_2,2,2,2_]Cl was the less thermally stable IL. The mass loss trends of the other five ILs were similar. However, the beginning temperatures of the mass loss were different. The mass of [N_8,8,8,1_]Cl lost firstly from 125°C and accelerated to nearly 99% at 250°C. So, the two quaternary ammonium ILs were the less stable ILs. The mass loss trends of [C_12_MIm]Br and [C_14_MIm]Br were nearly the same, losing quickly from ∼225°C and nearly 100% at 300°C. The thermal stabilities of the two typical IL surfactants were more than those of the two quaternary ammonium ILs. The mass loss of [HOEtMIm][BF_4_] began to accelerate from 250°C, while that for [HOEtMIm][NTf_2_] from 350°C. Both of the mass losses were nearly 100% at 600°C. Such results prove the most thermal stable of [HOEtMIm][BF_4_] and [HOEtMIm][NTf_2_].

Figure 3(b) shows the DTG results of the six ILs. It can be seen that the temperature of the maximum mass loss rate for different ILs was different. The temperature for [HOEtMIm][NTf_2_] was the highest (450°C), the second one was [HOEtMIm][BF_4_] (∼400°C), and the lowest two were 275°C for [N_2,2,2,2_]Cl and 200°C for [N_8,8,8,1_]Cl.

So, combining the TG and DTG results, the thermal stability of the ILs from strong to weak was [HOEtMIm][NTf_2_] > [HOEtMIm][BF_4_] > [C_12_MIm]Br > [C_14_MIm]Br > [N_2,2,2,2_]Cl > [N_8,8,8,1_]Cl.

### 3.4. Oxidation Results of the IL-Mixed Coals

**Figure 4:**
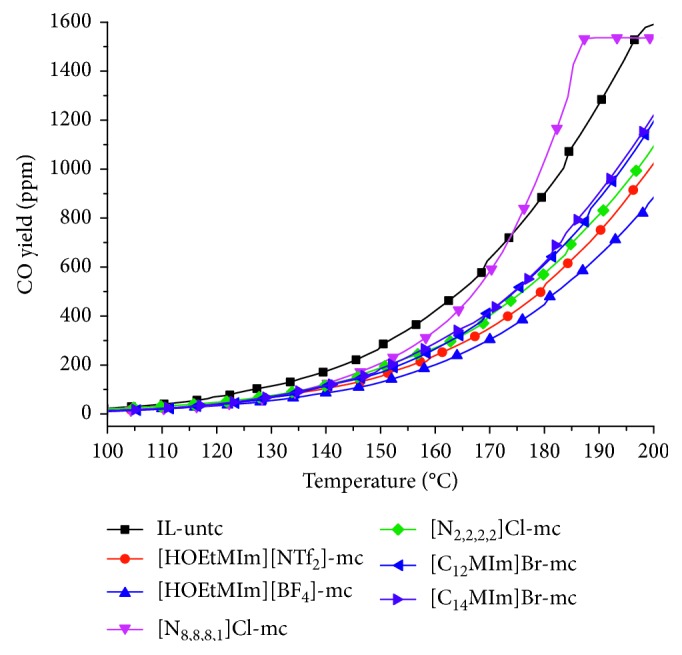
CO yield changes of different coals.

Figure 4 showed the gas product CO of the six IL-mixed coals by TP oxidation measurement. The CO yield of the IL-mixed coals was less than that of IL-untreated coal after 110°C except that of [N_8,8,8,1_]Cl-treated coal. The coal mixed with [N_8,8,8,1_]Cl produced more CO than coal unmixed with IL after ∼175°C, which was mainly caused by the CO production from the thermal decomposition of the IL [N_8,8,8,1_]Cl with bad thermal stability. The CO yields of the other five IL-mixed coals were less than those of IL-untreated coal, and the yield order from less to more was [HOEtMIm][BF_4_]-mc < [HOEtMIm][NTf_2_]-mc < [N_2,2,2,2_]Cl-mc < [C_12_MIm]Br-mc < [C_14_MIm]Br-mc < IL-untc.

**Figure 5:**
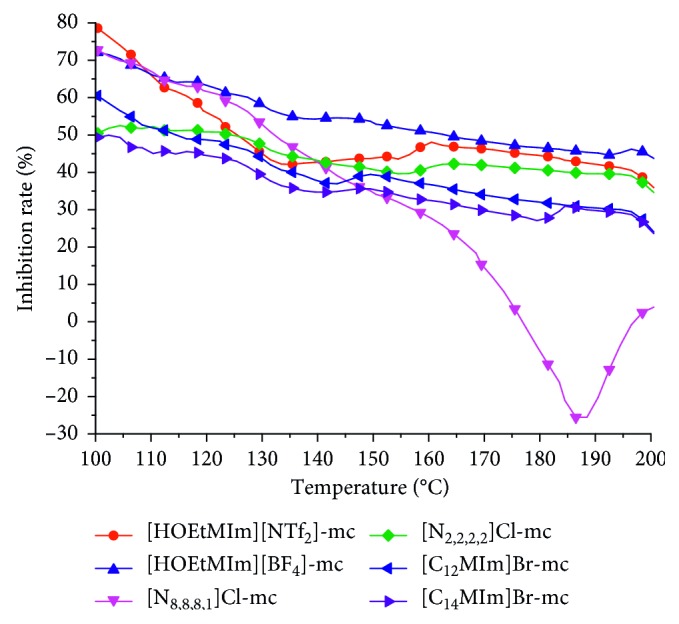
Inhibition rate curves with increasing temperature.

The inhibition rate of the ILs on coal oxidation can be calculated based on the formula *R*=((*A* − *B*)/*A*) × 100% [22, 23], where *R* is the inhibition rate (%), *A* is the CO yield of the IL-untreated coal (ppm), and *B* is the CO yield of the IL-mixed coals (ppm). The results are shown in Figure 5.

The inhibition rate results were mainly analyzed between 100 and 200°C because the significant change of CO product occurred from 100°C. From Figure 3, the inhibition rate for different ILs was different and the inhibition rate reduced with increasing temperature except that of [N_8,8,8,1_]Cl. The two highest inhibition rates at 100°C were from [HOEtMIm][NTf_2_] and [HOEtMIm][BF_4_]. The inhibition rate of [HOEtMIm][NTf_2_] was nearly 80% at 100°C, then reduced to ∼45% at 135°C and lastly stayed at ∼40%. The inhibition rate of [HOEtMIm][BF_4_] was nearly 75% at 100°C, then reduced with increasing temperature and was about 50% at the end of the experiment. The inhibition rates of [C_12_MIm]Br and [C_14_MIm]Br were 60% and 50% at 100°C, respectively. Then, the two rates reduced with the increasing temperature and both were ∼30% at 200°C. The inhibition rate of [N_2,2,2,2_]Cl changed from 50% at 100°C to 40% at 200°C. The [N_8,8,8,1_]Cl had a high inhibition rate at 100°C and then reduced until no inhibition effect at ∼175°C, indicating that the IL [N_8,8,8,1_]Cl accelerated the coal oxidation reactivity between 175 and 200°C.

In summary, the ILs with a higher inhibition rate and longer inhibition time were [HOEtMIm][BF_4_] and [HOEtMIm][NTf_2_], which also have better thermal stability.

### 3.5. Activation Energy Results of the IL-Mixed Coals

According to the Arrhenius formula, the activation energy can be calculated by the equation ln(*f*(*c*)/*T*^2^)=−(*E*/*RT*)+ln(*AR*/*βE*) [24, 25] when the reaction series is set as 1, where *E* is the apparent activation energy (J/mol), *R* is the gas constant (*R* = 8.314 J/(K·mol)), *T* is the coal sample temperature (K), *f*(*c*) = ln(*c*_0_/*c*) (*c*_0_ is the initial oxygen concentration, 21%; *c* is the oxygen concentration at temperature *T* (mol/cm^3^)), *A is* the former factor, and *β* is the heating rate (*β* = *dT*/*dt* = 274.15 K/min). The relationship between ln[*f*(*c*)/*T*^2^] and 1/*T* is linear so that the *y* = ln[*f*(*c*)/*T*^2^] and *x* = 1/*T* are set and the fitting result of *y*∼*x* can give intercept *γ* and slope *μ*. According to *μ* = −*E*/*R*, the apparent activation energy *E* = −*μR* can be obtained.

**Figure 6:**
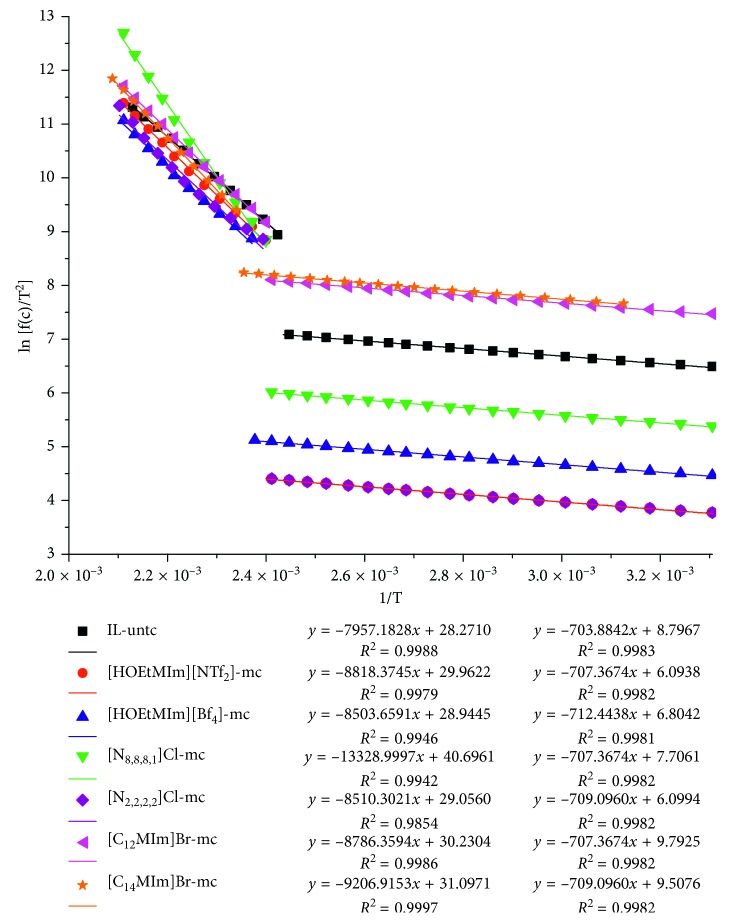
Linear fitting results of ln[*f*(*c*)/*T*^2^] to 1/*T* for different coal samples.

According to the linear relationship, the whole temperature range can be divided into two stages for fitting the data points, which is shown in Figure 6. According to the fitting results, the apparent activation energy values of IL-untreated coal and six IL-mixed coals can be obtained, which are shown in Table 3.

The activation energy represents the difficult degree of the oxidation reaction, where the greater the activation energy is, the more difficult the reaction is. The activation energy at the first temperature stage was generally small, which is because only easy reactions with small activation energies occurred [10, 26]. The activation energies of IL-mixed coals were slightly larger than those of IL-untreated coal, indicating the reaction for IL-mixed coals happened more difficultly. The maximum activation energy was for [HOEtMIm][BF_4_]-mixed coal. All the activation energies at the second temperature stage were larger than those at the first temperature stage, indicating the reactions with larger activation energies occurring. The activation energies of the IL-mixed coals were also larger than those of the IL-untreated coal in the second temperature stage, demonstrating the more difficult reaction for IL-mixed coals. The activation energy of [N_8,8,8,1_]Cl-mixed coal was far larger than that of the other coals, indicating the reaction with larger activation energy occurring. These reactions not only included the oxidation of coal but also the sharp reaction of [N_8,8,8,1_]Cl itself because of its bad thermal stability (Figure 3). As a result, the activation energy of [N_8,8,8,1_]Cl-mixed coal became larger and cannot be compared with the other five activation energies. The temperature at which the two temperature stages divided was 138°C for IL-untreated coal, while the dividing temperature for IL-mixed coals was delayed 4 to 10°C, indicating the beginning of the difficult reaction of coal was delayed. This was helpful for inhibiting coal oxidation. The delayed temperature for [HOEtMIm][BF_4_]-mixed coal was the maximum (10°C).

To sum up, all the ILs can increase the activation energy of coal. However, the poor thermal stability affected the results of [N_8,8,8,1_]Cl-mixed coal; hence, the other five ILs were suitable agents for inhibiting coal oxidation and the [HOEtMIm][BF_4_] and [C_14_MIm]Br may be the best ILs according to the delayed temperature and increased activation energies.

### 3.6. Functional Group Results of the IL-Treated Coals

**Figure 7:**
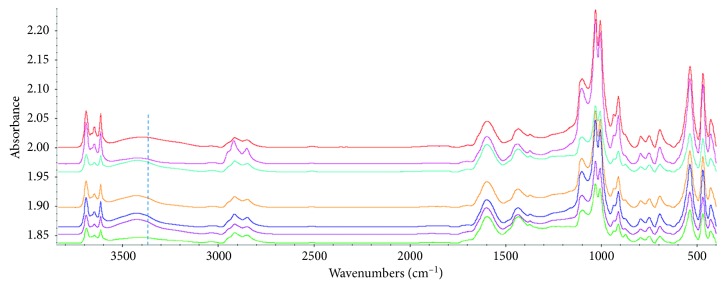
FTIR spectra of all coal samples (from top to bottom along the dotted line were the FTIR spectra of IL-untc, [N_8,8,8,1_]Cl-tc, [N_2,2,2,2_]Cl-tc, [C_12_MIm]Br-tc, [C_14_MIm]Br-tc, [HOEtMIm][BF_4_]-tc, and [HOEtMIm][NTf_2_]-tc).

Figure 7 shows the FTIR spectra of IL-untreated coal and IL-treated coals at room temperature at the Common Scale.

From Figure 7, the peaks of the main functional groups of all coal samples appeared, indicating that the main structure of coal was not changed. However, the absorption strength of some groups changed, such as the hydrogen bond interactions (3600–3100 cm^−1^), the aliphatic C-H stretching (3000–2800 cm^−1^), the ether bonds (1300–1000 cm^−1^), and the aromatic C-H bending (900–650 cm^−1^) [27]. The aromatic C=C stretching at 1600 cm^−1^ was nearly unchanged, indicating the polyaromatic system of coal was unaffected.

The hydrogen bonds associated by hydroxyl reduced remarkably in [N_8,8,8,1_]Cl- and [HOEtMIm][NTf_2_]-treated coals. For the other four IL-treated coals, the hydrogen bonds at low wavenumber (3300–3100 cm^−1^) decreased significantly. Such results indicated the decrease of the hydrogen bonds in IL-treated coals. The peaks of aliphatic C-H bonds only increased in [N_8,8,8,1_]Cl-treated coal because of the more aliphatic C-H bonds from residual IL [N_8,8,8,1_]Cl- in [N_8,8,8,1_]Cl-treated coal. The peak strength of the ether bonds reduced to different degrees in IL-treated coals except that in [N_8,8,8,1_]Cl-treated coal. The peak of aromatic C-H bending changed little. These results indicated the effect of IL on functional groups of coal.

**Figure 8:**
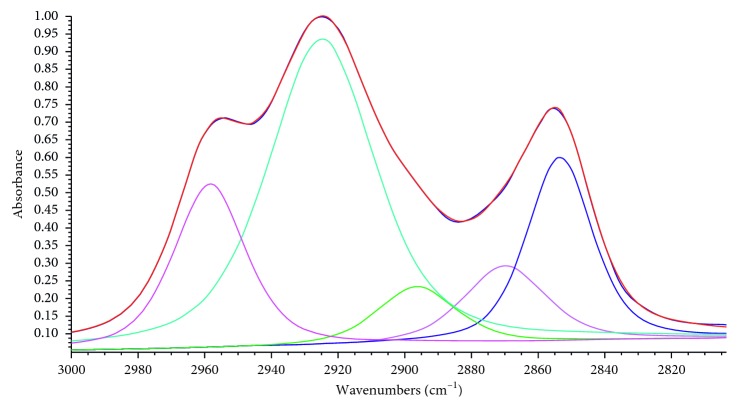
Fitting spectra of IL-untreated coal at 3000∼2800 cm^−1^ (the five fitting spectra at 2960, 2928, 2896, 2871, and 2854 cm^−1^ from left to right belong to methyl asymmetrical stretching vibration, methylene asymmetrical stretching vibration, methylene stretching vibration, methyl symmetrical stretching vibration, methylene symmetrical stretching vibration, respectively).

In order to quantitatively analyze these changes, the FTIR spectra were fitted and the peak area ratios of *A*_CH_2__/*A*_CH_3__ and *A*_ar_/*A*_al_ were chosen to characterize the change degree of coal structure. The two ratios were calculated by the equations *A*_CH_2__/*A*_CH_3__=*A*_(2853cm^−1^)_/*A*_(2918cm^−1^)_ and *A*_ar_/*A*_al_=*A*_(900 − 650cm^−1^)_/*A*_(3000 − 2800cm^−1^)_ [27]. The *A*_CH_2__/*A*_CH_3__ characterizes the length and degree of the aliphatic side chains and the compact of coal structure. The lower *A*_CH_2__/*A*_CH_3__ represents shorter aliphatic side chains in coal and more compact structure of coal. The *A*_ar_/*A*_al_ represents the aromaticity and reactivity of coal. The higher *A*_ar_/*A*_al_ represents higher aromatic degree and lower oxidation activity of coal [27, 28].  Figure 8 presents the fitting spectra of IL-untreated coal at 3000–2800 cm^−1^.  Table 4 shows the results of the *A*_CH_2__/*A*_CH_3__ and *A*_ar_/*A*_al_.

From Table 4, all the ratios of *A*_CH_2__/*A*_CH_3__ of IL-treated coals were lower than those of IL-untreated coals in different degrees, indicating the shorter aliphatic side chains and more compact structure of the IL-treated coals. The lowest ratios of *A*_CH_2__/*A*_CH_3__ were from [HOEtMIm][BF_4_]- and [C_14_MIm]Br-treated coals. The relatively high *A*_CH_2__/*A*_CH_3__ of [N_8,8,8,1_]Cl-treated coal was related with the residual IL [N_8,8,8,1_]Cl in [N_8,8,8,1_]Cl-treated coal, which resulted in more methylene groups in treated coal. All the *A*_ar_/*A*_al_ of the IL-treated coals were higher than that of IL-untreated coals except [N_8,8,8,1_]Cl-treated coal, indicating higher aromatic degree and lower oxidation activity of the five IL-treated coals. The maximum *A*_ar_/*A*_al_ was from [HOEtMIm][NTf_2_]-treated coal. The lower *A*_ar_/*A*_al_ of [N_8,8,8,1_]Cl-treated coal was also related with the more aliphatic methylene from residual of IL [N_8,8,8,1_]Cl in treated coal.

In conclusion, the ILs which can effectively destroy coal structure and decrease coal oxidation activity were [HOEtMIm][BF_4_], [HOEtMIm][NTf_2_], and [C_14_MIm]Br, while the IL with less destructive effect was [N_8,8,8,1_]Cl.

## 4. Conclusions

The effects of six IL surfactants on the oxidation characteristics and functional groups of coal were studied by means of critical micelle concentration, surface tension, thermogravimetric analysis, temperature-programmed oxidation, and FTIR measurements.

Firstly, the lower critical micelle concentration for the ILs except the [N_2,2,2,2_]Cl suggests the favorable surface activity of these ILs so that the ILs used can play both inhibitor and surfactant roles. The surface activity of the six IL solutions from strong to weak was [N_8,8,8,1_]Cl > [C_14_MIm]Br > [C_12_MIm]Br > [HOEtMIm][NTf_2_] > [HOEtMIm][BF_4_] > [N_2,2,2,2_]Cl. The thermal stability of the ILs from strong to weak was [HOEtMIm][NTf_2_] > [HOEtMIm][BF_4_] > [C_12_MIm]Br > [C_14_MIm]Br > [N_2,2,2,2_]Cl > [N_8,8,8,1_]Cl.

Secondly, the oxidation activity of IL-mixed coals was weakened to different degrees according to the CO yield except [N_8,8,8,1_]Cl. The poor thermal stability of IL [N_8,8,8,1_]Cl accelerated the coal oxidation reactivity between 175 and 200°C so that the other five ILs were suitable for inhibiting coal oxidation. The ILs [HOEtMIm][BF_4_] and [HOEtMIm][NTf_2_] had higher inhibition rate, longer inhibition time, and also better thermal stability. The activation energy results further confirmed the inhibition effect of the five ILs; particularly, the [HOEtMIm][BF_4_] and [C_14_MIm]Br may be the best ILs according to the delayed temperature and increased activation energies.

Lastly, the change of functional groups of coal showed that ILs can affect the hydrogen bonds, aliphatic groups, and aromatic groups. Such effect can inhibit coal oxidation activity. It was inferred that the [HOEtMIm][BF_4_], [HOEtMIm][NTf_2_], and [C_14_MIm]Br can effectively destroy coal structure and decrease coal oxidation activity combined with the quantitative analysis of the decrease of *A*_CH_2__/*A*_CH_3__ and the increase of *A*_ar_/*A*_al_.

## Figures and Tables

**Table 1:** CMC results of the six ILs.

ILs	CMC (mol/L)	CMC (g/L)
[HOEtMIm][NTf_2_]	0.001	0.407
[HOEtMIm][BF_4_]	0.01	2.140
[N_8,8,8,1_]Cl	0.002	0.808
[N_2,2,2,2_]Cl	0.8	132.568
[C_12_MIm]Br	0.008	2.651
[C_14_MIm]Br	0.004	1.438

**Table 2:** Surface tension of the ILs solutions.

Solutions	Distilled water	[HOEtMIm][NTf_2_]	[HOEtMIm][BF_4_]	[N_8,8,8,1_]Cl	[N_2,2,2,2_]Cl	[C_12_MIm]Br	[C_14_MIm]Br
Surface tension (mN/m)	72.55	36.29	42.71	28.53	50.96	35.80	34.21
Standard deviation	0.10	0.07	0.15	0.11	0.14	0.11	0.10

**Table 3:** Apparent activation energy of all coal samples at different temperature stages.

Coal samples	Temperature stage (°C)	Slope (*μ*)	Activation energy *E* (kJ/mol)
IL-untc	29∼138	−703.8842	5.852
	138∼202	−7957.1828	66.156
[HOEtMIm][NTf_2_]-mc	29∼142	−707.3674	5.881
	142∼202	−8818.3745	73.316
[HOEtMIm][BF_4_]-mc	29∼148	−712.4438	5.923
	148∼202	−8503.6591	70.699
[N_8,8,8,1_]Cl-mc	29∼142	−707.3674	5.881
	142∼202	−13328.9997	110.817
[N_2,2,2,2_]Cl-mc	29∼144	−709.0960	5.895
	144∼202	−8510.3021	70.755
[C_12_MIm]Br-mc	29∼142	−707.3674	5.881
	142∼202	−8786.3594	73.050
[C_14_MIm]Br-mc	29∼144	−709.0960	5.895
	144∼202	−9206.9153	76.546

**Table 4:** Results of the *A*_CH_2__/*A*_CH_3__ and *A*_ar_/*A*_al_ for different coal samples.

Coal sample	IL-untc	[HOEtMIm][NTf_2_]-tc	[HOEtMIm][BF_4_]-tc	[N_8,8,8,1_]Cl-tc	[N_2,2,2,2_]Cl-tc	[C_12_MIm]Br-tc	[C_14_MIm]Br-tc
*A*_CH_2__/*A*_CH_3__	0.680	0.591	0.537	0.671	0.589	0.545	0.537
*A*_ar_/*A*_al_	0.727	0.910	0.830	0.545	0.763	0.873	0.858

## Data Availability

The data in Figures 1–5 are used to support the findings of this study.
